# On‐line hemodiafiltration therapy for end‐stage kidney disease patients: Promises for the future? What's next?

**DOI:** 10.1111/sdi.13092

**Published:** 2022-05-05

**Authors:** Bernard Canaud, Andrew Davenport, Thomas A. Golper

**Affiliations:** ^1^ School of Medicine Montpellier University Montpellier France; ^2^ Global Medical Office FMC Deutschland Bad Homburg Germany; ^3^ University College London Department of Renal Medicine Royal Free Hospital, University College London UK; ^4^ Vanderbilt University Medical Center Nashville Tennessee USA

## Abstract

On‐line hemodiafiltration (OL‐HDF) is currently the most advanced form of blood purification modality leading convective‐based therapies in end‐stage kidney disease patients. By adding a high convective component to the diffusive clearance achieved with highly permeable dialyzers, OL‐HDF reinforces removal of small MWt compounds and enlarges the spectrum of uremic compounds cleared up to middle and large MWt compounds. The biological and clinical benefits of convective‐based therapy are currently also being explored in a revisited hybrid modality, combining an increased internal filtration process with a more open membrane. Regular use of ultrapure dialysis fluid required by convective‐based therapies improves the bio‐incompatibility of the extracorporeal circuit so reducing inflammatory responses. On‐line production of substitution fluid, relying on a cold sterilization by ultrafiltration, has several advantages: First, it is a safe and established process; and second, it provides an unlimited amount of substitution fluid at the same cost as regular ultrapure dialysis fluid. As such, OL‐HDF is adaptable to all substitution modalities (post, pre, or mixed‐HDF), thus allowing the dialytic convective dose to be adjusted to the individual patient needs. The development of OL‐HDF opens new pathways such as task automation simplifying care workflow. All these features make OL‐HDF the most versatile dialysis modality that can be now integrated in various treatment schedules according to session time and frequency (daily, nocturnal, or alternate day) or location (incenter, satellite, or potentially home‐based therapy).

## 1 BACKGROUND

On‐line hemodiafiltration (OL‐HDF) is the epitome of almost 30 years of technical development and clinical research into dialysis treatments. This series of monographs provide an updated in‐depth assessment of OL‐HDF compared to conventional high flux hemodialysis. In this comprehensive review, most fields of interest for clinicians have been addressed by renown international experts providing an OL‐HDF State of the Art in a practical and objective manner. In brief, they include the Medical Rationale for OL‐HDF, Basic Physics, Technical Prerequisite, Clinical Implementation and Monitoring, Clinical indications, Clinical and Biological Effects in Adults and Children, and Patient Outcomes and Value‐Based Healthcare Perspective.

Current state‐of‐the‐art technology and increasing numbers of patients treated worldwide confirm the absolute safety of OL‐HDF, the ease of implementation in multiple clinical settings, the superior efficacy of OL‐HDF compared to high flux HD in removing a larger molecular weight spectrum of uremic compounds and reducing the inflammatory state and vascular injury, and the immediate clinical benefits in terms of reducing intradialytic morbidity. In the long term, OL‐HDF has value‐based health care sustainable perspectives as indicated by its current acceptance by many different health care systems and has been reported to improve patient outcomes provided an adequate dialytic convective dose is delivered.

## 2 WHAT NEW INFORMATION IS BEING COLLECTED?

Two large randomized controlled trials (CONVINCE and H4RT) of more than 2800 patients are currently ongoing in Europe.^1^, ^2^ Although addressing some of the same questions, regarding patient outcomes (hard clinical endpoints), patient perception (patient‐reported outcomes and quality of life), and health care costs, they employ different tools and methods and will provide further clinical evidences within the next 2 to 3 years.

In addition, several clinical studies are exploring the potential benefits of the combination of increased internal filtration with a more open dialyzer membrane, supporting the growing interest for convective‐based therapies in the renal community.

## 3 WHAT ARE FUTURE PERSPECTIVES?

OL‐HDF remains a tool that has already brought advances in blood purification and already offers a paradigm shift in renal replacement therapy for patients with end‐stage kidney failure. However, as we have learned, renal replacement therapy for patients with chronic kidney disease has been a long journey that started some 60 years ago with improvements over time in its various components (buffer, membrane flux, treatment time, dialysate composition purity, and HD machine options) supported by new clinical and biological findings. This continuous journey will not stop with OL‐HDF.

Further developments are expected, coming either from more personalized treatment schedules (incremental, daily, nocturnal, and alternate day), or from dialyzers offering additional clearance of uremic toxins, in particular modifications to remove protein‐bound uremic toxins (adsorption and displacement), or from new dialysis machine functionality (biosensor, feedback loop control, and smart algorithms) or from integration of dialysis machines into a web‐based network system facilitating remote monitoring, control, and providing support for clinical decision‐making.

Success in renal replacement therapy is not a destination but rather a journey in which OL‐HDF represents a major step forward designed to improve patient perceptions and outcomes.

## CONFLICT OF INTEREST

No conflict of interests.
